# 2-AG and anandamide enhance hippocampal long-term potentiation *via* suppression of inhibition

**DOI:** 10.3389/fncel.2022.1023541

**Published:** 2022-09-21

**Authors:** Fouad Lemtiri-Chlieh, Eric S. Levine

**Affiliations:** Department of Neuroscience, University of Connecticut School of Medicine, Farmington, CT, United States

**Keywords:** endocannabinoid (eCB), hippocampus, LTP (long term potentiation), anandamide (AEA), 2-arachichodonyl glycerol, GABAergic inhibition

## Abstract

It is widely accepted that exogenous cannabinoids can impair short-term memory and cognition in humans and other animals. This is likely related to the inhibition of long-term potentiation (LTP), a form of synaptic plasticity, by the global and sustained activation of CB1 cannabinoid receptors in the presence of exogenous agonists. Conversely, the temporally and spatially restricted release of endogenous cannabinoid (eCB) ligands may enhance synaptic plasticity in a synapse-specific manner. We examined the role of eCB signaling in LTP by recording field excitatory postsynaptic potentials (fEPSPs) in the CA1 stratum radiatum in hippocampal slices from juvenile mice. LTP was induced either electrically, by theta burst stimulation (TBS), or pharmacologically, by treatment for 15 min with a solution designed to increase intracellular cAMP (chem-LTP). A stable and long-lasting potentiation in fEPSP slope following TBS was significantly reduced by blocking cannabinoid receptor activation with CB1 receptor antagonists. Chem-LTP caused a sustained 2-fold increase in fEPSP slope and was also blocked by CB1 receptor antagonists. TBS-LTP was partially reduced by inhibiting the synthesis of the endogenous ligands 2-arachidonylglycerol (2-AG) and anandamide. A similar effect was observed with chem-LTP. Blocking inhibitory synapses completely prevented the effect of CB1 receptor antagonists or inhibition of eCB synthesis on TBS-LTP and chem-LTP. These results indicate that simultaneous activation of CB1 receptors by 2-AG and anandamide enhances TBS-induced and pharmacologically-induced LTP, and this effect is mediated by the suppression of inhibition at GABAergic synapses.

## Introduction

It is widely accepted that exogenous cannabinoids can impair short-term memory and cognition in humans and other animals (Abush and Akirav, 2010). This is likely related at least in part to the inhibition of long-term potentiation (LTP), a form of synaptic plasticity, by the global and sustained activation of CB1 cannabinoid receptors by exogenous agonists (Auclair et al., 2000; Hoffman et al., 2007; Abush and Akirav, 2010). Conversely, the temporally and spatially restricted release of endogenous cannabinoid ligands may enhance synaptic plasticity in a synapse-specific manner (Carlson et al., 2002; Lin et al., 2011; Silva-Cruz et al., 2017). The functional roles of endocannabinoids (eCBs) are complex because they modulate synaptic transmission *via* suppression of GABA and glutamate release, with opposing effects on postsynaptic excitability (Trettel and Levine, 2003; Younts and Castillo, 2014; Yeh et al., 2017).

The two major endocannabinoids in the brain are 2-arachidonylglycerol (2-AG), the most abundant endocannabinoid ligand, and anandamide (AEA; Mechoulam and Parker, 2013). These endogenous ligands are synthesized postsynaptically, with diacylglycerol (DAG) lipase serving as the principal synthetic enzyme for 2-AG, and NAPE-PLD as a major synthetic enzyme for AEA (Sugiura et al., 2002; Basavarajappa, 2007). These endogenous ligands are thought to be released from postsynaptic sites and generally act in a retrograde manner at presynaptic CB1 receptors (Castillo et al., 2012; Kano, 2014). The VR1 capsaicin receptor and other receptors have also been proposed to mediate some of the effects of endocannabinoid signaling. The metabolic breakdown for 2-AG is mostly through monoacylglycerol (MAG) lipase, and fatty acid amide hydrolase (FAAH) is the major metabolic enzyme for AEA breakdown (Sugiura et al., 2002; Basavarajappa, 2007).

In the hippocampus, which is a key structure for learning and memory, CB1 receptors are expressed in both excitatory and inhibitory presynaptic terminals (Marsicano et al., 2003; Monory et al., 2015). Whereas the exogenous cannabinoid tetrahydrocannabinol can disrupt hippocampal LTP induction, other studies have shown that eCB signaling can enhance LTP through suppression of GABAergic inhibition (Carlson et al., 2002; de Oliveira Alvares et al., 2006). In the present studies, we examined the role of endocannabinoid signaling in both electrically-induced and pharmacologically-induced forms of hippocampal LTP.

## Methods

### Brain slice preparation

All animal procedures were conducted according to protocols approved by the University of Connecticut Health Center Institutional Animal Care and Use Committee. Postnatal day 15–31 Swiss CD-1 mice (Charles River, Wilmington, MA) were decapitated under 3.5% isoflurane anesthesia and the brains were harvested quickly and placed into an ice-cold cutting solution composed of (in mM): 125 NaCl, 2.5 KCl, 1.25 NaH_2_PO_4_, 25 NaHCO_3_, 0.05 CaCl_2_, 2 MgCl_2_, 10 glucose, carboxygenated with 95% O_2_–5% CO_2_ (pH 7.3 and osmolality 305 ± 5 mmol·kg^−1^). Longitudinal slices (350 μm) containing the hippocampus were cut using a vibratome (Leica VT1000S, Buffalo Grove, IL). The slices were placed in a homemade chamber containing an incubation solution at a temperature of 34–35°C for 20 min before being transferred to room temperature for at least another 60–90 min before recording. The incubation solution was similar in composition to the cutting solution but with 2 CaCl_2_ and 4 MgSO_4_ (in place of 0.05 CaCl_2_ and 2 MgCl_2_). Slices were then individually transferred to a recording chamber (Warner Instruments, Harvard Bioscience, Inc.) fixed to the stage of an upright microscope (Olympus BX50WI, Allentown, PA) fitted with a 4× objective (Olympus PLN Plan Achromat). During recordings, slices were continuously perfused at 2 ml/min with artificial cerebrospinal fluid (aCSF) consisting of (in mM) 125 NaCl, 2.5 KCl, 1.25 NaH_2_PO_4_, 25 NaHCO_3_, 2 CaCl_2_, 1.3 MgCl_2_, and 10 glucose (pH 7.3 and osmolality 305 ± 5 mmol·kg^−1^). pH was equilibrated by continuous bubbling with 95% O_2_–5% CO_2_.

### Electrophysiology

Hippocampal field excitatory postsynaptic potentials (fEPSPs) were recorded at room temperature from the stratum radiatum layer of CA1. Micropipettes were pulled from borosilicate glass capillaries (Flaming/Brown P-97 micropipette puller; Sutter Instrument, Novato, CA) and filled with aCSF yielding resistances between 4 and 6 MΩ. To stimulate the axons emanating from the CA3 region, a bipolar tungsten electrode (1 MΩ; WPI, Sarasota, FL) was placed in the Schaffer collateral pathway approximately 250 μm lateral to the recording micropipette. fEPSPs were evoked every 20 s (0.05 Hz) using a baseline intensity that evoked less than half the maximal response, as determined from the input/output curves carried out before each experiment. Long-term synaptic potentiation (LTP) was induced either electrically by theta-burst stimulation (TBS-LTP) or chemically (chem-LTP). The TBS induction protocol consisted of one train of 10 bursts (each burst contained five stimuli at 100 Hz) delivered at 5 Hz. Chem-LTP was induced by perfusing the slices for 15 min with a modified aCSF (induction cocktail) containing the forskolin (50 μM) and rolipram (0.1 μM) in the presence of low Mg (0.05 mM). This chemical LTP induction protocol has been used in other studies (Otmakhov et al., 2004; Grey and Burrell, 2008).

### Chemicals

Unless otherwise stated, all drugs were from Tocris Biosciences/Bio-techne (Minneapolis, MN, USA) and were delivered by bath perfusion. Drugs were first prepared as concentrated stock solution insolvents and stored at −20°C. The stock solutions were dissolved in 100% dimethyl sulfoxide (DMSO) and the final concentration of DMSO did not exceed 0.1%. Drug stock solutions were diluted into aCSF on the day of recording to the final concentrations: forskolin (50 μM), rolipram (0.1 μM), picrotoxin (50 μM), capsazepine (10 μM), (*RS*)-CPP (3 μM), and APV (50 μM). NESS 0327 (1 μM), LEI-401 (10 μM), and SR141716A (10 μM) were obtained from Cayman Chemical Company (Ann Arbor, MI, USA) and DO34 (1 μM) was obtained from Sigma-Aldrich (St. Louis, MO, USA).

### Data analysis

Offline analysis of the fEPSPs data was carried out using both Clampfit (Molecular Devices, CA) and Prism 7.03 (GraphPad Software, San Diego, CA). The peak of the fEPSPs was measured as the difference between the end of the fiber volley signal and the fEPSP peak response while the fEPSP slopes were generated from linear regressions of the rising phase (20%–80% of the peak response). Group data are reported as mean ± SE. Statistical comparisons were made using one-way ANOVA and Dunnett’s multiple comparison test.

## Results

### Blocking CB1 receptors impairs both TBS-LTP and chem-LTP

**Figure 1:**
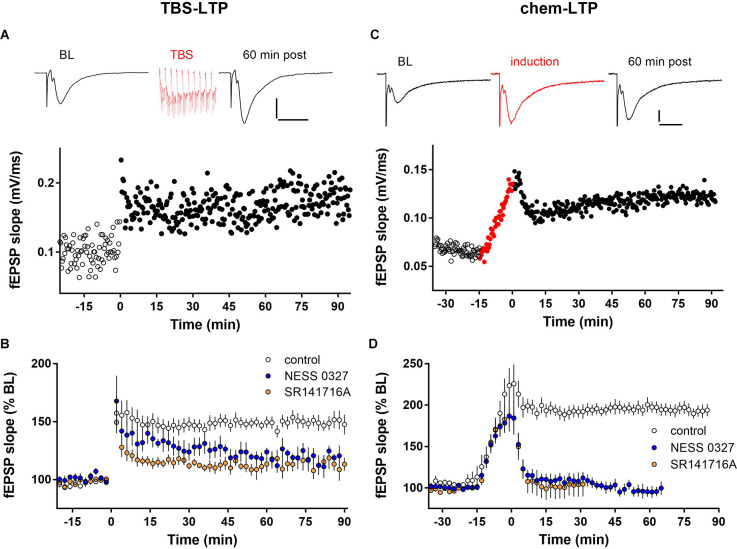
LTP expression is inhibited by CB1 receptor blockade. **(A)** Individual example of LTP induced by one train of TBS containing 10 bursts delivered at minute 0. Top: Example fEPSP sweeps from baseline (BL) and 60 min post-TBS. Scale bars: 0.2 mV/20 ms. **(B)** Group data for TBS-LTP under control conditions (*n* = 10) or in the presence of the CB1 receptor blockers NESS 0327 (*n* = 7) or SR141716A (*n* = 6). **(C)** Individual example of chem-LTP induced by 15 min exposure to the induction cocktail (red symbols, -15–0 min). Top: Example sweeps from baseline (BL), during induction, and 60 min post-induction. Scale bars: 0.2 mV/20 ms. **(D)** Group data for chem-LTP under control conditions (*n* = 12) or in the presence of the CB1 receptor blockers NESS 0327 (*n* = 8) or SR141716A (*n* = 6).

**Figure 2:**
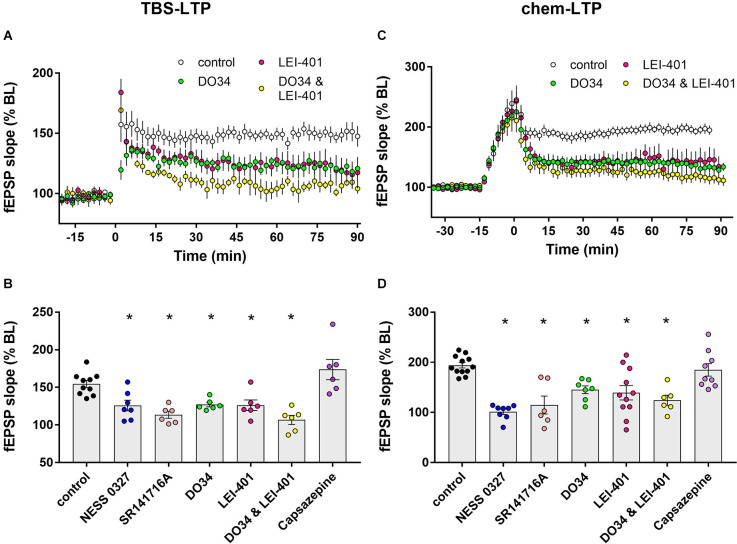
Blocking eCB synthesis impairs LTP expression.** (A)** Example time courses of TBS-LTP induced by one train of TBS delivered at minute 0 in the presence or absence of eCB synthesis inhibitors. **(B)** Group data for the magnitude of TBS-LTP at 60 min under various conditions. Ordinary one-way ANOVA, *F*_(8,53)_ = 14.52, *p* < 0.0001. *p < 0.05 compared to control by Dunnett’s multiple comparison test. **(C)** Example time courses of chem-LTP induced by 15 min exposure to the induction cocktail (−15–0 min) in the presence or absence of eCB synthesis inhibitors. **(D)** Group data for the magnitude of chem-LTP at 60 min under various conditions. Ordinary one-way ANOVA, *F*_(8,62)_ = 9.1, *p* < 0.0001. *p < 0.05 compared to control by Dunnett’s multiple comparison test.

We examined the role of eCB signaling in LTP by recording fEPSPs in the CA1 stratum radiatum in hippocampal slices from juvenile mice. As described above, LTP was induced either electrically, by one train of TBS (TBS-LTP), or pharmacologically, by treatment for 15 min with an induction cocktail containing the adenylyl cyclase activator forskolin and the phosphodiesterase inhibitor rolipram in the presence of low Mg (chem-LTP). In response to TBS stimulation, significant potentiation (~50% increase of fEPSP slope compared to baseline) was induced by a single train of TBS and lasted at least 90 min. Changes in fEPSP slope were paralleled by changes in peak fEPSP amplitude. An individual example of LTP induced by a single train of TBS is shown in Figure 1A. There was no change in the size of the presynaptic fiber volley after LTP induction, indicating that the increase in fEPSP magnitude is not due to a change in presynaptic axonal excitability (Supplementary Figure 1A). As shown in the group data in Figures 1B and 2B, TBS-induced LTP caused an increase of fEPSP slope to 154.5% ± 4.7% of baseline at 60 min post-induction (*n* = 10). The magnitude of this potentiation was significantly reduced by preventing cannabinoid receptor activation with the selective CB1 receptor competitive antagonist NESS-0327 (Figures 1B, 2B; 125.8 ± 7.1% of baseline, *n* = 7). To confirm the effect of CB1 receptor blockade, we repeated this experiment using the CB1 receptor inverse agonist SR-141716A, which blocked TBS-induced LTP to a similar extent as NESS-0327 (Figures 1B, 2B, 113.5% ± 4.5% of baseline, *n* = 6). Neither NESS-0327 nor SR-141716A had any effect on baseline fEPSP amplitude or slope before LTP induction, and there was no effect of administration of the DMSO vehicle alone (Supplementary Figure 1B). TBS-LTP was dependent on NMDA receptor activation, as it was completely blocked in the presence of the NMDA receptor antagonists CPP or APV (Supplementary Figure 1B).

Exposure to the chem-LTP induction cocktail typically caused a greater than 2-fold increase by the end of the 15 min exposure period, and a maintained ~2-fold increase in fEPSP slope for up to 90 min, as shown in the representative example in Figure 1C. As shown in the group data in Figures 1D and 2D, chem-LTP resulted in an average increase to 193.9% ± 5.4% of baseline (*n* = 12) for greater than 60 min. This LTP was completely blocked by the CB1 receptor antagonist NESS-0327 (101.4% ± 5.3% of baseline, *n* = 8, Figures 1D, 2D), although the increase in fEPSP slope was still apparent during the induction period (Supplementary Figure 1C). We repeated this experiment with SR-141716A, which blocked chem-LTP to a similar extent as NESS-0327 and also did not block the increase during the induction period (114.8% ± 18.0% of baseline, *n* = 6, Figures 1D, 2D). There was no effect of administration of the vehicle alone (Supplementary Figure 1D). NMDA receptor activation may also contribute to chem-LTP, as this potentiation appeared to be partially inhibited in the presence of the NMDA receptor antagonists CPP or APV, but this effect was not statistically significant (Supplementary Figure 1D). Because endogenous cannabinoids can also activate the VR1 receptor, we examined the effects of the VR1 receptor antagonist capsazepine, which did not affect TBS-induced LTP (173.7% ± 13.6% of baseline, *n* = 6; Figure 2B) nor chem-LTP (184.7% ± 12.3% of baseline, *n* = 9; Figure 2D).

### 2-AG and anandamide signaling contribute to TBS-LTP and chem-LTP

Impaired LTP after blocking CB1 receptors suggests that endogenously-released cannabinoids normally enhance LTP. We next attempted to identify the eCB ligand(s) that are involved in LTP modulation. The synthesis of the endogenous ligand 2-AG was inhibited using the DAG lipase inhibitor DO34 (Wilkerson et al., 2017) and anandamide synthesis was targeted using the NAPE-PLD inhibitor LEI-401 (Castellani et al., 2017; Mock et al., 2020). As shown in Figures 2A,B, the magnitude of TBS-LTP was reduced by ~50% by inhibiting 2-AG synthesis with DO34. A similar reduction in LTP magnitude was seen by inhibiting AEA synthesis with LEI-401, indicating that both 2-AG and AEA contribute to eCB modulation of TBS-LTP. Interestingly, D034 and LEI-401 combined appeared to have an additive effect on TBS-LTP, but this was not statistically significant (106.8% ± 6.0% of baseline, *n* = 6, Figures 2A,B). Neither D034 nor LEI-401 had any effect on baseline fEPSP magnitude before LTP induction. In a parallel set of experiments shown in Figures 2C,D, chem-LTP was partially inhibited by DO34 and LEI-401 individually, but had no significant additive effect, indicating that 2-AG and AEA also contribute to the eCB modulation of chem-LTP.

### Endocannabinoid-mediated enhancement of LTP requires GABAergic transmission

**Figure 3:**
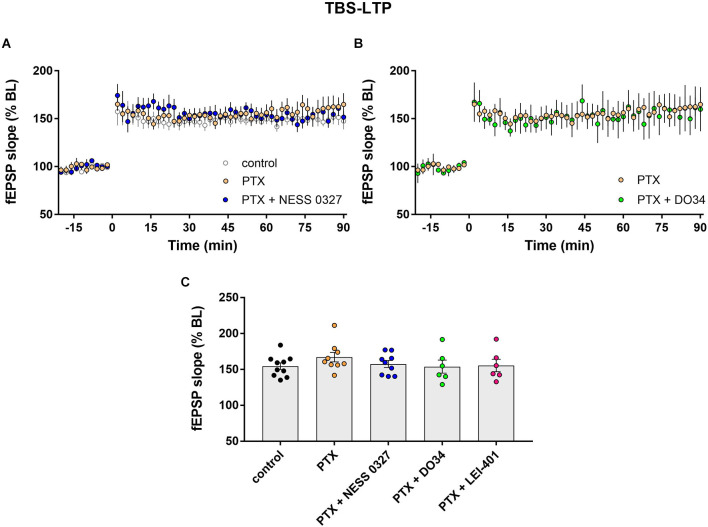
eCB modulation of TBS-LTP requires GABAergic transmission. **(A)** Example time courses of TBS-LTP induced by one train of TBS delivered at minute 0 in control conditions or in the presence of picrotoxin (PTX) or PTX plus the CB1 receptor antagonist NESS 0327. **(B)** Example time courses of TBS-LTP induced by one train of TBS in the presence of PTX or PTX plus the DAG-lipase inhibitor DO34. **(C)** Group data for the magnitude of TBS-LTP at 60 min post-induction under various conditions. Control data repeated from Figure 2.

Impaired LTP after CB1 receptor blockade or inhibition of eCB synthesis suggests that activation of CB1 receptors by endogenous 2-AG and anandamide normally enhances the magnitude of LTP. We thus hypothesized that the predominant eCB effect is due to eCB-mediated suppression of inhibition. We, therefore, examined TBS-LTP while blocking inhibitory synapses with the GABA_A_ receptor blocker picrotoxin (PTX). The addition of PTX alone had no significant effect on LTP (Figures 3A,C; *n* = 9). Consistent with our hypothesis, PTX completely prevented the inhibition of LTP by the CB1 receptor antagonist NESS 0327, indicating that intact GABAergic transmission is required for the eCB effect (Figures 3A,C; *n* = 9). The presence of PTX did not unmask any enhancing effect of CB1 receptor blockade, suggesting minimal eCB-mediated suppression of glutamate release under these conditions. PTX also prevented the effect of inhibiting eCB synthesis, as DO34 and LEI-401 did not affect TBS-LTP in the presence of PTX (Figures 3B,C; *n* = 6 for each condition).

**Figure 4:**
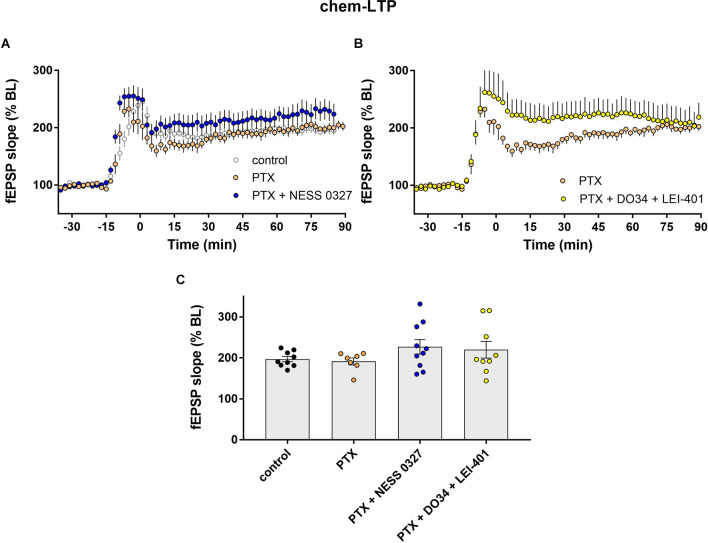
eCB modulation of chem-LTP requires GABAergic transmission.** (A)** Example time courses of chem-LTP in control conditions or in the presence of picrotoxin (PTX) or PTX plus the CB1 antagonist NESS 0327. **(B)** Example time courses of chem-LTP in the presence of PTX or PTX plus the eCB synthesis inhibitors DO34 and LEI-401. **(C)** Group data for the magnitude of chem-LTP at 60 min post-induction under various conditions. Control data repeated from Figure 2.

We next examined chem-LTP under the same experimental conditions as described above, i.e., in the presence of PTX and other eCB blocking/modulating drugs. The results were comparable to what we described for TBS-LTP. The addition of PTX alone did not affect the induction nor the maintenance of chem-LTP (Figures 4A,C; *n* = 7) but prevented the inhibition of LTP by the CB1 receptor antagonist NESS 0327 (Figures 4A,C; *n* = 10). Inhibition of chem-LTP by the combination of DO34 and LEI-401 (Figures 4B,C; *n* = 9) was also blocked by PTX. These results indicate that endogenous 2-AG and anandamide activation of CB1 receptors enhance TBS-LTP and chem-LTP, and this effect requires intact GABAergic transmission.

## Discussion

In the present study, we examined the role of eCB signaling in LTP by recording fEPSPs in the CA1 stratum radiatum in hippocampal slices from juvenile mice. LTP was induced either electrically, by theta burst stimulation, or pharmacologically, by treatment for 15 min with a combination of forskolin and rolipram in the presence of low magnesium. TBS-LTP lasted greater than 60 min and the magnitude of this potentiation was significantly reduced by blocking cannabinoid receptor activation with the selective CB1 receptor antagonist NESS-0327 or the inverse agonist SR-141716A. Chem-LTP caused a sustained two-fold increase in fEPSP slope and this potentiation was also blocked by CB1 receptor antagonists. Because endogenous cannabinoids can also activate the VR1 receptor, we examined the effects of the VR1 antagonist capsazepine, which had no effect on TBS-LTP or chem-LTP. We next attempted to identify the eCB ligand(s) that are involved in LTP modulation. The synthesis of the endogenous ligand 2-AG was inhibited using the DAG lipase inhibitor DO34 and anandamide synthesis was targeted using the NAPE-PLD inhibitor LEI-401. TBS-LTP was inhibited by ~50% by LEI and DO34 individually. Similarly, chem-LTP was partially inhibited by DO34 and LEI-401 individually and had a greater effect when combined. These results indicate that endogenously-released 2-AG and AEA enhance the magnitude of TBS-LTP and chem-LTP *via* CB1 receptor signaling.

Endocannabinoids act as negative feedback regulators of presynaptic transmitter release at both excitatory and inhibitory synapses in the hippocampus. Selective deletion of CB1 in glutamatergic or GABAergic neurons can facilitate, or depress, LTP respectively (Monory et al., 2015). Different LTP-inducing stimuli may selectively engage eCB signaling at either or both types of synapses. We hypothesized that activation of CB1 receptors by endogenous 2-AG and AEA enhanced LTP due to eCB-mediated suppression of inhibition. We, therefore, examined the effect of blocking inhibitory synapses with the GABA_A_ receptor blocker PTX. PTX completely prevented the effect of CB1 antagonists or inhibition of eCB synthesis on TBS-LTP and chem-LTP, thus suppression of GABAergic inhibition prevents impairment of LTP by the inhibition of eCB signaling. This indicates that intact GABAergic transmission is required for eCB modulation. The presence of PTX did not unmask any enhancing effect of CB1 receptor blockade, suggesting minimal eCB-mediated suppression of glutamate release under these conditions. These results indicate that 2-AG and AEA enhance TBS-induced and pharmacologically-induced LTP through signaling at inhibitory synapses. Interestingly, TBS-LTP was completely dependent on NMDA receptor activation, whereas chem-LTP was not significantly inhibited by NMDA receptor antagonists, suggesting that eCB modulation of these forms of LTP is not dependent on NMDA receptor involvement.

Many studies of eCB modulation of LTP and learning implicate 2-AG as the major endogenous agonist (Pan et al., 2011; Schurman et al., 2019), but manipulating AEA levels also modulates synaptic plasticity as well as learning and memory tasks (Ade and Lovinger, 2007; Lin et al., 2011; Subbanna et al., 2013; Yang et al., 2014; Zimmermann et al., 2019). Importantly, the present studies suggest that both AEA and 2-AG can be released simultaneously by LTP-inducing stimuli to enhance potentiation *via* suppression of GABAergic inhibition. TBS can also induce long-term depression at inhibitory synapses (Younts et al., 2013; Younts and Castillo, 2014; Zhao et al., 2015) which may contribute to the facilitation of LTP. Under the present conditions, eCB signaling appeared to be biased towards inhibitory synapses, but this may vary depending on several factors, including developmental age, brain region, and cell type. Different induction protocols may also affect the synaptic specificity of eCB signaling and may produce differential effects on 2-AG vs. AEA release. Understanding the specificity of eCB signaling and the differential effects of neuronal activity on AEA and 2-AG mobilization will be critical in unraveling their contributions to synaptic plasticity, learning, and memory.

## Data Availability Statement

The raw data supporting the conclusions of this article will be made available by the authors, without undue reservation.

## Ethics Statement

The animal study was reviewed and approved by Institutional Animal Care and Use Committee, University of Connecticut School of Medicine.

## Author Contributions

FL-C conducted experiments and carried out data analysis. EL and FL-C designed the study and wrote the manuscript. All authors contributed to the article and approved the submitted version.

## Funding

This work was supported by National Institutes of Health.
