# Supplementary material for: 2-AG and anandamide enhance hippocampal long-term potentiation via suppression of inhibition
Source: Front Cell Neurosci. 2022 Sep 21;16:1023541. doi: 10.3389/fncel.2022.1023541 (PMC9534525; doi:10.3389/fncel.2022.1023541)
Supplement: Supplementary file 1 [file Table_1.DOCX]

Supplementary Material


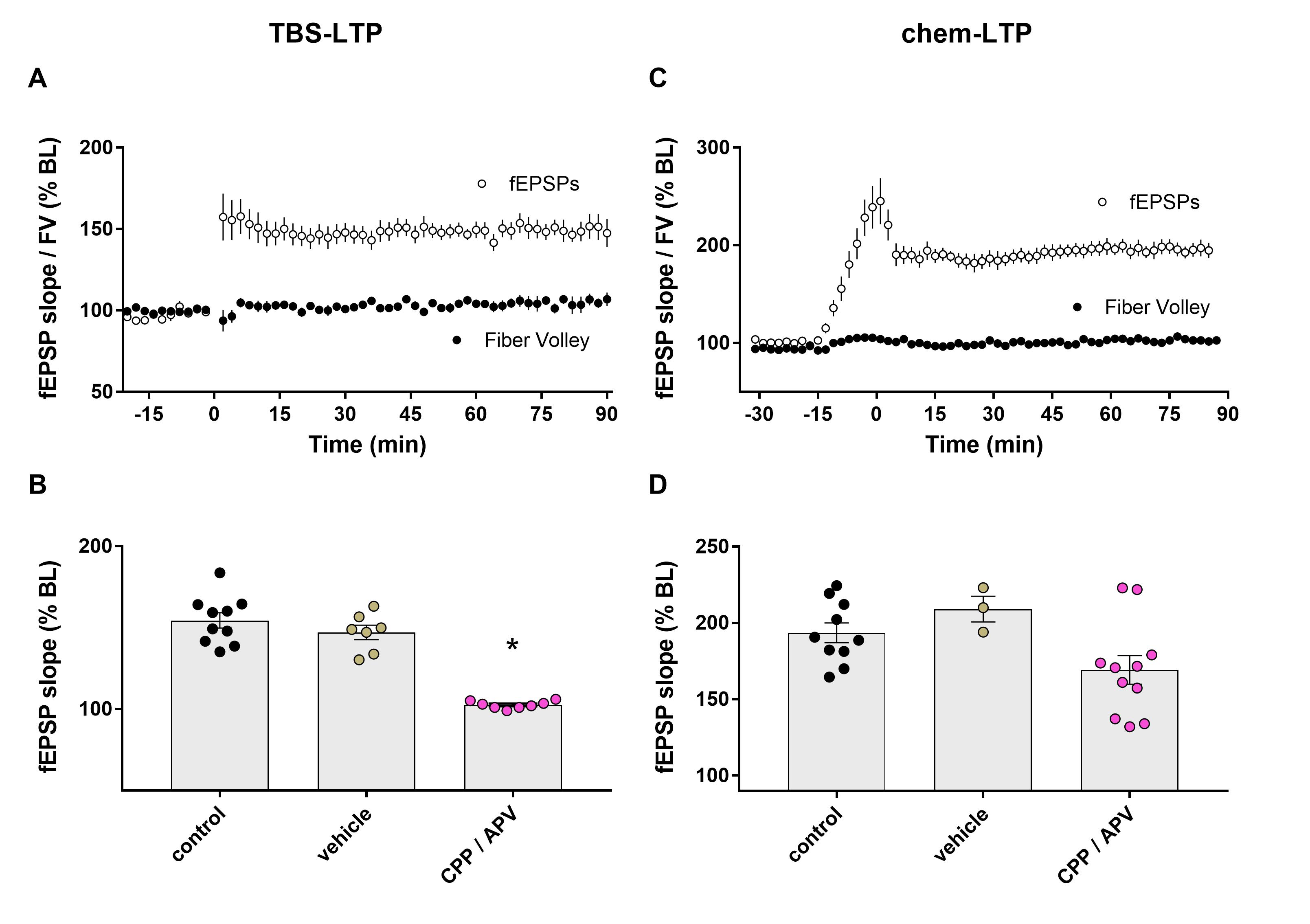


**Supplementary Figure 1.** **A)** Group time course of the fEPSP slope and fiber volley amplitude following TBS delivered at minute 0 (n=10). **B)** Effects of DMSO vehicle (n=7) or NMDA receptor antagonists (3 µM CPP or 50 µM APV, n=8) on the magnitude of TBS-LTP at 60 min post-induction. *, p<0.05 compared to control. Control data repeated from Figure 2. **C)** Group time course of the fEPSP slope and fiber volley amplitude following chem-LTP cocktail delivered from -15-0 min (n=12). **D)** Effects of vehicle (n=3) or NMDA receptor antagonists (n=11) on the magnitude of chem-LTP at 60 min post-induction. Control data repeated from Figure 2.
